# Using Digital Phenotyping for Depression Screening in Community-Dwelling Older Adults: Bayesian Multilevel Hurdle Model Machine Learning Approach

**DOI:** 10.2196/69494

**Published:** 2026-05-15

**Authors:** Moo-Kwon Chung, Hyo-Sang Lim, Sang Yup Lee, Hyo Seok Baek, Jinhee Lee, Kyoung Joung Lee, Taeksoo Shin, Min-Hyuk Kim, Sangwon Hwang, Erdenebayar Urtnasan, Ji Young Park, Dan Hee Kwon, Jin-kyung Lee

**Affiliations:** 1 Department of Global Public Administration, Yonsei University Mirae Campus, Wonju, Republic of Korea; 2 Division of Software, Yonsei University Mirae Campus, Wonju, Republic of Korea; 3 Department of Communication, Yonsei University Sinchon Campus, Seoul, Republic of Korea; 4 Department of Computer Science, Yonsei University Mirae Campus, Wonju, Republic of Korea; 5 Department of Psychiatry, Yonsei University Wonju College of Medicine, Wonju, Republic of Korea; 6 Office of the President, Songho University, Hoengseong, Gangwon-do, Republic of Korea; 7 Department of Business Administration, Yonsei University Mirae Campus, Wonju, Republic of Korea; 8 Department of Precision Medicine, Yonsei University Wonju College of Medicine, Wonju, Republic of Korea; 9 Division of AI Semiconductor, Yonsei University Mirae Campus, Wonju, Republic of Korea; 10 Department of Social Welfare, Sangji University, Wonju, Republic of Korea; 11 Department of Health Administration, Yonsei University Mirae Campus, Wonju, Republic of Korea; 12 Health IT Center, Gachon University Gil Hospital, 773, Namdong-daero, Namdong-gu, Incheon, 21556, Republic of Korea, 82 32 460 8090

**Keywords:** digital phenotyping, depression, older adults, machine learning, Bayesian modeling, multilevel modeling

## Abstract

**Background:**

With the rapidly aging population, mental health among older adults has received growing attention. Although the likelihood of experiencing depressive symptoms is higher in late adulthood, older adults are more reluctant to visit a clinic due to the stigma surrounding mental health issues, and many remain undiagnosed and untreated. Digital phenotyping has emerged as a promising approach to mitigate this problem. Longitudinal monitoring via wearable devices can facilitate the timely identification of depressive symptoms in older adults. However, there has not been sufficient investigation to develop a machine learning approach that accounts for between-person and within-person characteristics.

**Objective:**

This study aimed to investigate the utility of active and passive digital phenotyping data collected via wearable devices for monitoring the probability and severity of depressive symptoms. Specifically, we applied multilevel hurdle modeling within a machine learning framework to enable efficient depression screening in the general population, with a focus on community-dwelling older adults.

**Methods:**

We analyzed 1011 cases reported by 147 older Korean adults for 2 years. Participants were asked to complete the 9-item Patient Health Questionnaire (PHQ-9) items in our mobile app during the last week of each month. In addition to the annual in-person data collection, we also collected active and passive sensing data from participants via smartphones and smartwatches. For dimensionality reduction on 44 features, parallel analysis and principal component analysis were used. With the extracted 6 principal components (PCs), a Bayesian multilevel hurdle model was used.

**Results:**

When constructing PCs, the weekly stress rating from active data and sleep-related features from passive data were the top 5 contributing features. Among the 6 PCs, the PC consisting of low psychological distress and high social support was significantly associated with depressive symptoms in community-dwelling older adults. This Bayesian multilevel hurdle model showed good performance in screening for depressive symptom severity (*R*^2^=0.53) and in distinguishing between those with and without symptoms (area under the receiver operating characteristic curve=0.88 and *F*_1_-score=0.75) on the test data. The between-person variance was larger than the within-person variance, especially in explaining the probability of depressive symptoms.

**Conclusions:**

In mental health screening, active and passive digital phenotyping data can be used in conjunction with traditional clinical screening tools to monitor depressive symptoms among community-dwelling older adults. Dimensionality reduction via parallel analysis and principal component analysis can help identify latent risk profiles. Given the nested data structure and heterogeneity in depressive symptoms, a Bayesian multilevel hurdle model within a machine learning framework may be helpful for depression screening. Overall, digital phenotyping can be a useful tool for personalized, within-person health tracking, even after accounting for substantial between-person variance. We recommend future work to address data imbalance to further strengthen this approach.

## Introduction

Depression is a highly prevalent mental health disorder, with approximately 5% of the global population affected by this disease [1]. Depression can be considered dangerous because it can lead to suicidal ideation when it is not appropriately treated in a timely manner [1]. Despite its high prevalence, many people with depressive symptoms neither recognize their need for medical treatment nor receive appropriate medical care [2,3]. Within this group, one particularly vulnerable segment of society is older adults. In the developmental stage, late adulthood is the period of a higher likelihood of experiencing depressive symptoms [4]. However, this population tends to have a strong taboo against seeing psychiatrists for their mental health [5,6]. Consequently, many older adults with depressive symptoms are likely to remain undiagnosed and untreated, which can increase their mortality and other health problems [7].

Compared to many other physical health problems that are objectively screened by physical biomarkers, many mental health problems, such as depressive disorder, are diagnosed based exclusively on a client’s subjective statements about experiencing symptoms [8,9]. Unless the patient is open and willing to undergo treatment, it is much harder to detect depressive symptoms and prescribe an appropriate treatment regimen at the onset of illness. The recent innovation of digital phenotyping has been receiving increasing global attention due to its potential to remotely monitor and screen for early signals of depressive symptoms [9]. One of the biggest benefits of digital phenotyping is its ability to reduce recall bias, a prevalent issue in traditional screening methods. Traditional depression screening tools are limited by recall bias because they rely solely on retrospective survey measures. By using smart devices, digital phenotyping enables the collection of in situ data on participants’ mood, activity, sleep, and other behaviors [10]. With digital phenotyping, it is possible to monitor real-time data regarding depressive symptoms and to identify individuals experiencing depressive symptoms promptly [8]. Since digital phenotyping can enable real-time monitoring in natural settings, advances in smartphone technology have spurred academic interest in this area in psychiatry [8]. By applying digital phenotyping to real-time monitoring, it is possible to reach out to those in need for early intervention. It would be helpful to identify individuals with depressive symptoms in the general population who may otherwise remain undetected.

In terms of the types of data collected in digital phenotyping, there are mainly 2 categories: active data and passive sensing data [8]. Active data includes self-report survey responses collected through smartphone apps, and passive sensing data includes moment-by-moment data collected unintentionally by sensors (eg, step counts or sleep log data collected via smart wearable devices) [9]. Active data is beneficial for understanding people’s subjective perceptions of their moods, stress, and life experiences; however, one drawback of this method is that it requires significant human effort to respond to screening questionnaires. Passive sensing data, on the other hand, enables real-time status monitoring [10] with easy access and minimal user input, which has attracted attention in psychiatry, particularly in studies on depression [11]. However, collecting passive sensing data often requires additional expensive digital devices (eg, smartwatches). Interestingly, despite the large number of theoretical frameworks, there have been relatively few empirical studies that build machine learning models using both active and passive digital phenotyping features. This might be because collecting digital phenotyping features from a large sample requires abundant funding and resources. Although smartphones are prevalent, the features of digital phenotyping that can be collected only by smartphones are limited, which can result in a modest sample size. The potential cost burdens that may arise when incorporating digital phenotyping into practice settings pose a risk that significantly diminishes its benefits for monitoring individuals experiencing depressive symptoms, especially in socioeconomically disadvantaged communities or those with limited access to medical services. Using a Bayesian machine learning approach, this study aims to explore how digital phenotyping features collected by smartphones and smartwatches can be used to screen for depressive symptoms among community-dwelling older adults. By doing this, we aim to examine how we can leverage active and passive digital phenotyping features despite the challenge of a moderate sample size.

In this study, one careful methodological consideration is the data structure accumulated in real-time monitoring. Given the nested longitudinal structure of the data, collected features should be treated differently at at least 2 levels: time-varying and time-invariant covariates. Despite the rapid increase in academic interest in machine learning algorithms following the rise of artificial intelligence, many machine learning studies have been based on cross-sectional designs. There has not been sufficient consideration of how to handle longitudinal data collected over different time points. To handle nested data appropriately, this study will combine a machine learning approach with a 2-level Bayesian multilevel model comprising within-person and between-person levels.

Expanding on the Bayesian multilevel model using digital phenotyping data, this study uses a hurdle model. Digital phenotyping enables easy data collection from the general population to screen for depressive symptoms. When screening a general population for depressive symptoms longitudinally, various depressive symptom trajectories are often reported. Although there is no consensus on how many trajectories exist or how these trajectory patterns appear, it is commonly reported that there is a significant proportion of those who have a stable low probability of depression, and that there are qualitatively heterogeneous groups experiencing different levels of depressive symptoms and varying levels of changes in depressive symptoms [12-14]. Those who have experienced depressive symptoms in the past are more vulnerable to depressive symptoms in the future [15]. Furthermore, those who have subthreshold depressive symptoms tend to experience more fluctuations in depressive symptoms over time [16]. It is common to see a large proportion of zeros in depressive symptoms when analyzing depression trajectories with longitudinal data from the general population. Since those without depressive symptoms differ qualitatively from those with depressive symptoms, applying a single regression or classification model to the entire dataset may not yield accurate estimates of feature importance. To address this issue, the zero-inflated model has been widely used for 9-item Patient Health Questionnaire (PHQ-9) data. However, when considering the theoretical assumption of sampling zeros, a hurdle model would be more appropriate to apply than the zero-inflated model [17]. In the zero-inflated model, structural zeros are treated differently from sampling zeros. For example, sampling zeros include cases where none are obtained despite trying, while structural zeros are cases of never trying. However, for the PHQ-9, a total score of 0 indicates no depressive symptoms. In other words, sampling zeros exactly reflect structural zeros. In a hurdle model, all sampling zeros are assumed to indicate true structural zeros [17,18]. Thus, this study will use a Bayesian multilevel hurdle model to examine the presence and severity of depressive symptoms in community-dwelling older adults. To better explain the associations between covariates and the target outcome variable, a hurdle model estimates 2 regression equations [17,18]. In the binary part, the hurdle model uses logistic regression to estimate the probability of having any depressive symptoms vs none [17,18]. In the continuous part, the hurdle model estimates linear regression coefficients for the severity of depressive symptoms among those with nonzero depressive symptom scores [17,18].

In summary, this study aims to investigate how active and passive digital phenotyping data collected from wearable devices can help monitor depressive symptoms in community-dwelling older adults using a Bayesian hurdle model with 2-level longitudinal data within a machine learning framework.

## Methods

### Procedure

To develop a machine learning algorithm for depressive symptoms in older adults, we recruited 685 Korean adults in their 50s to 80s residing in Wonju, a large city with both urban and rural areas in South Korea. The inclusion criteria were voluntary participation in this study, being 55 years of age or older, having no cognitive impairments, no alcohol or substance use disorders, no physical disabilities, and being able to concentrate throughout the 1.5-hour baseline interview. Data from 2 participants were excluded because they were identified as duplicates. Of 683 older adults who visited our campus and completed a one-on-one interview with our trained researchers, 411 participants agreed to monitor their depressive symptoms longitudinally and to install our smartphone app, developed exclusively for this research. Through in-person baseline data collection, participants reported their demographic characteristics, physical health, and psychological functioning using traditional survey tools. Soon after, we developed a smartphone app to monitor the depressive symptoms of the participants, which invited the participants to report their daily mood, weekly stress exposure, and monthly depressive symptoms. These app-based surveys collected active data over approximately 24 months (from March 2021 to March 2023) through smartphones. In total, 4566 cases reported by 352 respondents were included in the analysis, which included in-person annual screening data and active data (ie, mobile app survey responses), such as monthly PHQ-9 scores. For passive sensing data, however, the sample size became smaller due to the limited number of available smartwatches for the research. Our smartphone app, designed for daily, weekly, and monthly mobile app surveys, was also linked to the Samsung Health app, allowing step counts and sleep log data collected from smartwatches to be sent to our server through collaboration. The study protocol was published in an international, peer-reviewed medical journal [19]. In this study, we analyzed 1011 cases collected via smartwatches from 147 participants, including active and passive sensing data alongside traditional in-person survey tools.

### Measures

Subjective depressive symptoms were measured by PHQ-9 [20] within our smartphone app. In this study, we used 2 types of depressive symptoms as independent outcome variables. To investigate the association between digital phenotyping and depressive symptom severity, we first used the total PHQ-9 score as the outcome for the continuous part. In addition, to explore the potential application of our model for screening individuals with depressive symptoms in the general population, we also classified structural zeros vs nonzeros for the outcome in the binary part of our model.

Features extracted from active data collection from the participants’ reports through the smartphone app included the average and SD of the daily mood score, which ranged from very good “(1)” to very depressed “(5)” during a month, the average score and its SD of weekly stress exposure during a month, the frequency that a participant was exposed to stress related to their job, the frequency that a participant was exposed to stress related to interpersonal relationships, the frequency that a participant was exposed to stress related to major life events (ie, death, divorce, marriage, or birth), the frequency that a participant was exposed to stress related to health problems, the frequency that a participant was exposed to stress related to financial issues, and the frequency that a participant was exposed to stress related to extraordinary traumatic life events (eg, a crime, a natural disaster, or an accident) during each month.

Features extracted from passive sensing data collection included the average and SD of daily step counts during a month, the average duration (in minutes) spent in deep sleep, the average duration (in minutes) spent in light sleep, and the average duration (in minutes) spent in rapid eye movement (REM) sleep during daily sleep within a month, the average duration (in minutes) and SD of the first nonawake sleep stage after getting into bed, the average duration (in minutes) and SD of the last nonawake sleep stage before waking up, the efficacy of daily sleep, the frequency of awakening longer than 5 minutes during a night, and the difference between the average sleep duration on a weekday vs the average sleep duration on a weekend. All features were standardized before running the analyses.

In addition, the month variable and 3 categorical variables for seasons were also time-varying features, but we treated them differently. Given our assumption that depression is not linearly changing over time, we added 3 binary variables to reflect the 4 seasons. Given that summer has the longest period of sunlight, we set summer as the reference season, and other seasons (spring, fall, and winter) were coded as binary variables. After that, we tested the direct associations between the month variable or the 3 categorical variables and depressive symptom outcome variables. We found a significant relationship with the month variable, but we did not detect any significant direct relationships between the 3 seasonal binary variables and any outcome variable. Thus, we included only the month variable as the time variable in the final Bayesian model. For the 3 seasonal binary variables, we included them in the principal component analysis (PCA) with other features, but we did not include them separately from the principal components (PCs) in the final Bayesian model.

As constant features which were unchanging over time, demographic characteristics included participants’ sex (1: male; 0: female), age, education, average monthly income, the number of family members in a household, marital status (1: married and living with a spouse; 0: else), and whether a participant worked in agriculture (1: yes; 0: no). The monthly income was log-transformed because it was nonnormal. To screen participants’ physical and mental health conditions, features were included such as whether a participant regularly exercised (1: yes; 0: no), whether a participant had a history of smoking (1: yes; 0: no), how many alcoholic drinks a participant consumed in a month, how many hours a participant slept in a day, how many chronic diseases a participant had experienced in their lifetime, whether a participant had ever been diagnosed with major depressive disorder at a clinic, how many depressive episodes a participant had ever experienced in their lifetime based on the Mini-International Neuropsychiatric Interview (MINI) [21], the degree of generalized anxiety disorder based on the total score of the 7-item Generalized Anxiety Disorder scale (GAD-7) [22], the degree of loneliness based on the total score of the 20-item University of California Los Angeles Loneliness Scale (UCLA Loneliness) [23], the degree of perceived social support based on the total score of the 12-item Multidimensional Scale of Perceived Social Support (MSPSS) [24], and the number of types of early childhood traumas based on the total score of the 27-item Early Trauma Inventory–Short Form (ETI-SF) [25].

### Statistical Analysis

As a machine learning approach, we split the data at the observational level chronologically for each participant into 80% for training and 20% for testing. During data preprocessing, we examined all features from in-person, active, and passive sensing data and used Multiple Imputation by Chained Equations (MICE) to impute missing values separately to avoid data leakage. Of 1011 cases in total, 5 (0.49%) cases were missing from the daily mood surveys, and 22 (2.18%) cases were missing from the weekly stress surveys within the active data. Also, 11 (1.09%) cases did not have an answer about the number of depressive episodes in one’s lifetime in the in-person data. On the other hand, passive sensing data were largely complete, except for step counts. Because passive sensing data were collected at the millisecond level while depressive symptoms were assessed monthly, it was hard to observe missing data in passive sensing. In this study, we transformed all active and passive sensing features to the month level. Regarding step counts, 157 (15.53%) cases were treated as missing due to a technical transmission issue. That is, smartwatch-recorded step counts were not consistently transmitted to the server, whereas smartphone-recorded step counts were accurately captured. Upon identifying this issue during participant house visits, the collected step count data during this period were treated as missing to avoid the use of inaccurate values. Accordingly, MICE was applied to address these technically induced missing values, assuming that missingness was unrelated to depressive symptoms or physical activity behaviors. When imputing missing data among 43 features using MICE, we included the ID and time to preserve the longitudinal data structure. After that, to reduce feature dimensionality, PCA was performed. Using the recipes workflow in R, variables were first standardized to a mean of 0 and an SD of 1. The number of PCs to retain was determined using parallel analysis and the Cattell scree test. For PCA, we conducted a parallel analysis to determine the number of PCs for data reduction. In the PCA stage, using the recipe function, we calculated the means and SDs of the parameters from the training data and computed the PCA rotation. We applied these parameters to both the training and test data.

When we developed the machine learning algorithm, we used Bayesian modeling in R, considering our sample size. Unlike frequentist methods such as traditional linear or logistic regression models, which produce a single value for each estimate, Bayesian models use a posterior distribution by Markov Chain Monte Carlo sampling for each model parameter. By accounting for model uncertainty, a Bayesian model is known to produce more accurate estimates, and its benefits are even more pronounced when the sample size is modest. We used 4 chains, 4000 iterations, 4 cores, and 2000 warmup iterations. To account for the longitudinal data structure, with repeated measurements from participants, we used Bayesian multilevel modeling with the “*brms*” package and included a random intercept for individual ID. Finally, we used the hurdle model to account for the presence and severity of symptoms. By using 2 separate equations, such as regression in the continuous part and logistic regression in the binary part, the hurdle model aims to explain the probability of having depression and the severity of depressive symptoms effectively. Model coefficients were estimated using the training data, and we evaluated predictive performance and generalizability on independent test data. The model evaluation metrics for the continuous part, when the outcome is the total depressive symptom score, included *R*^2^, root-mean-square error (RMSE), and mean absolute error (MAE). Model evaluation metrics for the binary part, when the outcome is the probability of having depression, included the area under the receiver operating characteristic curve (AUC-ROC), precision, sensitivity, specificity, and *F*_1_-score. In the Bayesian multilevel hurdle model with 2 types of outcome variables, each parameter was reported with a regression coefficient, 95% credible intervals (CrIs), R-hat, bulk effective sample size (bulk-ESS), and tail-ESS. For the model diagnostics of the Bayesian multilevel hurdle model, we assessed model adequacy using posterior predictive checks, comparing observed data with replicated datasets drawn from the posterior predictive distribution. We also conducted Pareto smoothed importance sampling leave-one-out (PSIS-LOO) cross-validation. The reliability of the PSIS-LOO approximation was assessed using Pareto k diagnostic values.

### Ethical Considerations

Before collecting the data, the Institutional Review Board of the Yonsei University Mirae Campus (IRB no 1041849‐202401-SB-020-11) reviewed and approved all the procedures and measures involving human participants in this research. All participants provided written informed consent to participate in this study. All obtained data were deidentified. Participants received 30,000 won (approximately US $21.29) after completing the baseline in-person survey and received 10,000 Won (approximately US $7) every 3 months based on their completion rates in mobile app surveys. In this paper, no identification of individual participants is included in any text, images, or tables.

## Results

### Attrition Analysis Results

Among 411 participants who installed our smartphone app for data collection exclusively for research purposes, depressive symptoms (PHQ-9) and active data (eg, daily mood and weekly stress) were collected through the app from 352 participants. Passive sensing data (eg, daily step counts and sleep logs in milliseconds) were collected from 147 participants using a Samsung Galaxy smartwatch in addition to our mobile app. The sample characteristics of the participants in our datasets are presented in Multimedia Appendix 1. As shown in Multimedia Appendix 2, logistic regression was conducted to assess whether attrition bias was associated with demographic characteristics and health-related information. Regarding attrition for passive sensing data, there was no noticeable difference in all characteristics between 147 participants with smartwatches and 205 participants without smartwatches. Further, we tested for attrition bias in the outcome variables; however, no group difference was found due to attrition in the active and passive digital phenotyping data (*t*_350_=0.94; *P*=.35 for the severity of depressive symptoms and *t*_350_=−0.13; *P*=.89 for the probability of experiencing depressive symptoms).

### Parallel Analysis and PCA Results for Dimensional Reduction

**Figure 1.**
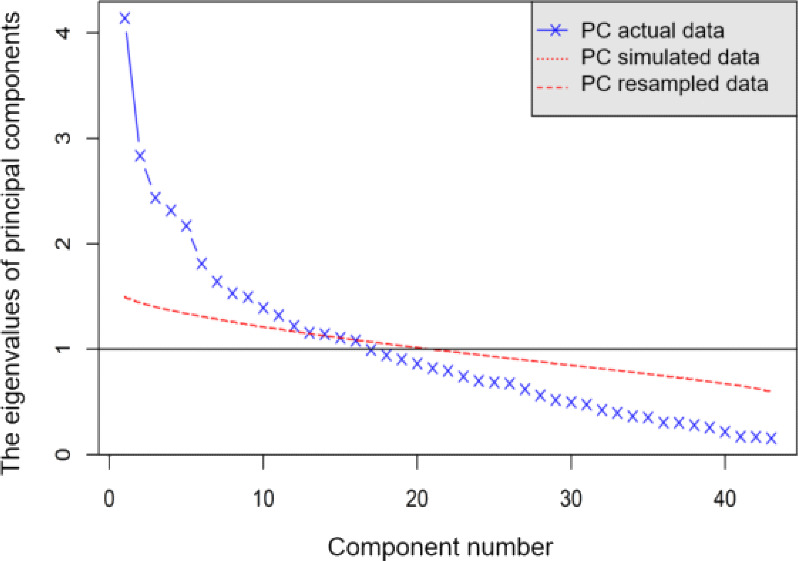
Parallel analysis scree plot for determining the number of principal components (PCs). Blue X marks on the line indicate eigenvalues of PCs produced from the observed dataset. A red dotted line indicates the average eigenvalues from randomly generated datasets. A red long-dashed line indicates eigenvalues from bootstrapped samples. In this plot, the red dotted line and the red long-dashed line overlap.

To reduce the feature dimensionality, we first conducted parallel analysis and PCA. Figure 1 shows the scree plot from the parallel analysis, and Table 1 shows the PCA results with eigenvalues greater than 1.0 retained. While the Kaiser criterion (eigenvalues >1) suggested 16 components, the parallel analysis results indicated that 12 components significantly exceeded the eigenvalues of a random dataset. However, we needed to reduce the number of PCs given the total sample size, and the scree plot showed that the slope flattened after the sixth component (Figure 1). We ultimately retained 6 components for interpretability, accounting for 36.53% of the total variance (Table 1).

**Table 1.** Principal component analysis results.

Principal components (n)	Eigenvalue	Percent of total variance explained	Cumulative percent of total variance explained
1	4.14	9.62	9.62
2	2.83	6.59	16.22
3	2.44	5.67	21.88
4	2.32	5.39	27.27
5	2.17	5.05	32.32
6	1.81	4.22	36.53
7	1.64	3.82	40.35
8	1.53	3.56	43.91
9	1.49	3.48	47.38
10	1.39	3.24	50.62
11	1.32	3.07	53.69
12	1.22	2.84	56.53
13	1.16	2.69	59.22
14	1.14	2.66	61.88
15	1.11	2.58	64.45
16	1.08	2.52	66.97

Table 2 shows the factor loadings of the top 5 contributing features for 6 PCs. For PC1, anxiety (λ=0.32), social support (λ=−0.32), loneliness (λ=0.29), the average of daily negative mood (λ=0.27), and the average weekly stress (λ=0.27) had higher factor loadings. This PC represented a dimension of psychological stress and low social support. The eigenvalue of PC1 was 4.14, which explained 9.62% of the total variance. For PC2, the top 5 contributing features were participants’ gender (λ=0.40 for males), smoking (λ=0.40), sleep time in the light sleep stage (λ=0.27), sleep time in the deep sleep stage (λ=0.24), and education level (λ=0.23). This PC reflected a high-sleep, educated, male smoker profile. It had an eigenvalue of 2.83 and explained 6.59% of the total variance. Combining participants’ demographic and psychological characteristics, PC3 reflected another profile. To be specific, income (λ=0.36), the average weekly stress (λ=0.28), SD of weekly stress (λ=0.26), and education level (λ=0.24) had positive factor loadings, while loneliness (λ=−0.26) had negative factor loadings for this PC. This PC reflected a high socioeconomic status, high-stress, and low-loneliness profile. It had an eigenvalue of 2.44 and explained 5.67% of the total variance. For PC4, light sleep minutes (λ=−0.33), REM sleep minutes (λ=−0.33), and deep sleep minutes (λ=−0.31) had higher factor loadings in a negative direction, whereas smoking (λ=0.25) and male (λ=0.25) had positive factor loadings. PC4 reflected a profile of a male smoker with low sleep duration. This PC had an eigenvalue of 2.32 and explained 5.39% of the total variance. For PC5, SD of sleep onset time (λ=0.29), working in agriculture (λ=0.28), and married status (λ=0.27) had positive factor loadings, while drinking (λ=−0.33) and total sleep hours (λ=-0.21) had negative factor loadings. This PC reflected a farmer’s profile with irregular sleep. It had an eigenvalue of 2.17 and explained 5.05% of the total variance. For PC6, higher factor loadings were found when participants had a higher education level (λ=0.39), walked less (λ=−0.34), had a higher income (λ=0.30), had more family members (λ=0.30), and had greater loneliness (λ=0.26). This PC reflected a high socioeconomic status, low-activity, and high-loneliness profile. It had an eigenvalue of 1.81 and explained 4.22% of the total variance.

**Table 2.** Top 5 contributing features for the 6 principal components.

Component and top 5 features	Loading
Principal component 1	
Anxiety	0.32
Social support	−0.32
Loneliness	0.29
Mean of daily negative mood	0.27
Mean of weekly stress	0.27
Principal component 2	
Male	0.40
Smoking	0.40
Light sleep minutes	0.27
Deep sleep minutes	0.24
Education	0.23
Principal component 3	
Income	0.36
Mean of weekly stress	0.28
SD of weekly stress	0.26
Loneliness	−0.26
Education	0.24
Principal component 4	
Light sleep minutes	−0.33
REM^a^ sleep minutes	−0.33
Deep sleep minutes	−0.31
Smoking	0.25
Male	0.25
Principal component 5	
Drinking	−0.33
SD of sleep onset time	0.29
Employed in agriculture	0.28
Married status	0.27
Total sleep hours	−0.21
Principal component 6	
Education	0.39
Mean of daily step counts	−0.34
Income	0.30
Family size	0.30
Loneliness	0.26

a REM: rapid eye movement.

### Bayesian Multilevel Hurdle Model for the Continuous Part: Total Score of Depressive Symptoms in Older Adults

In this continuous part, the outcome variable was the severity of depressive symptoms measured by the original PHQ-9 total score, ranging from 0 to 27. Table 3 shows the model results, including a regression coefficient for each PC. Among the 6 PCs, the first (*γ*=0.15, 95% CrI 0.08‐0.23) and the fourth (*γ*=0.09, 95% CrI 0.01‐0.18) were significantly associated with depressive symptom severity among community-dwelling older adults. That is, the severity of depressive symptoms was likely to be higher when a community-dwelling older adult experienced greater psychological distress and lower social support (PC1). Also, the severity of depressive symptoms was likely to be higher when an older adult had shorter REM, light, and deep sleep and was male with greater smoking. A significant variance in the intercepts (variance 0.435, 95% CrI 0.24‐0.76) indicates differences in depressive symptom severity across individuals. However, no significant trend over time was found (*γ*=−0.02, 95% CrI –0.03 to 0.00). R-hat measures whether the Markov Chain Monte Carlo chains have converged to the same posterior distribution. All R-hat values for the parameters were 1.00, indicating excellent convergence. Bulk-ESS shows whether the posterior mean is reliable, and tail-ESS shows whether CrIs are reliable. Bulk-ESS greater than 1000 and tail-ESS greater than 1000 are regarded as excellent. Bulk-ESS and tail-ESS were large enough, ranging from 3162 to 6267.

**Table 3.** Bayesian multilevel hurdle model estimates for the depressive symptoms.

Depressive symptom	Estimate (95% CrI^a^)	Estimate error	R-hat	Bulk-ESS^b^	Tail-ESS^c^
Fixed effects					
Continuous part					
Intercept	0.78 (0.51 to 1.04)	0.14	1.00	5544	5945
PC1^d^	0.15 (0.08 to 0.23)	0.04	1.00	3327	4148
PC2	0.01 (–0.08 to 0.10)	0.05	1.00	3162	4615
PC3	–0.09 (–0.18 to 0.00)	0.05	1.00	3378	4694
PC4	0.09 (0.01 to 0.18)	0.04	1.00	3647	5729
PC5	0.03 (–0.07 to 0.14)	0.05	1.00	4391	5859
PC6	0.05 (–0.05 to 0.15)	0.05	1.00	4286	5348
Month	–0.02 (–0.03 to 0.00)	0.01	1.00	8307	6267
Binary part					
Intercept	–1.36 (–2.29 to –0.47)	0.46	1.00	2817	4779
PC1	–0.79 (–1.12 to –0.49)	0.16	1.00	2792	4282
PC2	0.07 (–0.29 to 0.43)	0.18	1.00	2336	3958
PC3	–0.18 (–0.51 to 0.15)	0.17	1.00	2851	4578
PC4	–0.22 (–0.52 to 0.08)	0.15	1.00	3379	4768
PC5	–0.09 (–0.43 to 0.24)	0.17	1.00	3359	5312
PC6	0.05 (–0.31 to 0.41)	0.18	1.00	3850	5927
Month	0.11 (0.06 to 0.17)	0.03	1.00	6808	5793
Random effects					
SD (intercept: continuous part)	0.66 (0.49 to 0.87)	0.1	1.00	2821	4134
SD (intercept: binary part)	3.07 (2.31 to 4.06)	0.44	1.00	2803	4710

a CrI: credible interval.

b Bulk-ESS: bulk effective sample size.

c Tail-ESS: tail effective sample size.

d PC: principal component.

### Bayesian Multilevel Hurdle Model for the Binary Part: Identification of the Presence of Depressive Symptoms in Older Adults

In the Bayesian multilevel hurdle model for the probability of depression, the binary part modeled the probability that an observation belonged to the structural-zero group, while the continuous part modeled the outcome magnitude among observations capable of generating nonzero values. For the binary part, we used the same 6 PCs as in the continuous part, based on the rule of 10 and the scree plot pattern. The Bayesian multilevel hurdle model results for the binary part are presented in Table 3. Regarding the logistic regression coefficients for each parameter, the first PC (*γ*=−0.79, 95% CrI –1.12 to –0.49) was negatively associated with the outcome. In this binary part, there was a trend of increasing zeros over time (*γ*=0.11, 95% CrI 0.06‐0.17). All R-hat values for the parameters were 1.00, and bulk-ESS and tail-ESS were large enough, ranging from 2336 to 5927. The SD of the random intercepts for the binary outcome was 3.07 (95% CrI 2.31‐4.06).

### Model Diagnostics of the Bayesian Multilevel Hurdle Model

In the continuous part, although model performance decreased in the test set, the overall predictive accuracy remained acceptable, indicating reasonable generalizability. As shown in Table 4, the *R*^2^ was 0.650, the RMSE was 1.60, and the MAE was 0.95 for the training data. Compared to these, in the test data, the *R*^2^ was 0.53, the RMSE was 2.25, and the MAE was 1.22. Although the model evaluation metrics on the test data tended to be lower than those on the training data, it is promising to see that the Bayesian multilevel hurdle model explained 53% of the total variance when applied to new, unseen data from the participants. Of course, there was a 12% performance gap between the training and test data, indicating unexplained “noise” or secondary factors not yet captured by the current PC set, while the core drivers are identified.

**Figure 2.**
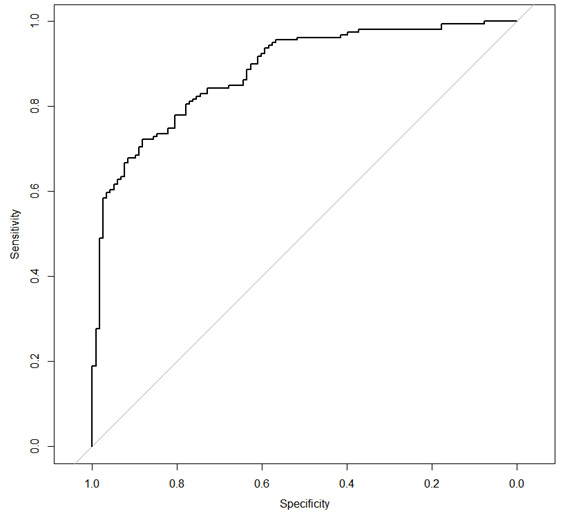
Receiver operating characteristic (ROC) curve for the probability of experiencing depressive symptoms in the test dataset using the Bayesian multilevel hurdle model (area under the curve [AUC]=0.88).

As performance metrics in the binary part (Table 4), the AUC-ROC was 0.95, the accuracy was 0.87, the precision was 0.86, the recall was 0.88, the specificity was 0.87, and the *F*_1_-score was 0.87 on the training data. For the test data, the AUC-ROC was 0.88, the accuracy was 0.79, the precision was 0.76, the recall was 0.75, the specificity was 0.82, and the *F*_1_-score was 0.75. Figure 2 shows the ROC curve and area under the curve for screening for susceptibility to experiencing depressive symptoms in the test dataset. The actual zeros were 534 (52.82%) of 1011 data in total. In the 8:2 split data, the actual zeros were 159 (59.11%) cases out of 269 data in the test dataset. Predicted zeros were also 161 (59.85%) out of 269 cases. Out of 161 (59.85%) cases, 30 (11.15%) cases of predicted zeros were incorrect, whereas 131 (48.70%) cases were correct. The model demonstrated strong discrimination on the training data and maintained good predictive performance on the independent test data, suggesting no evidence of severe overfitting.

**Figure 3.**
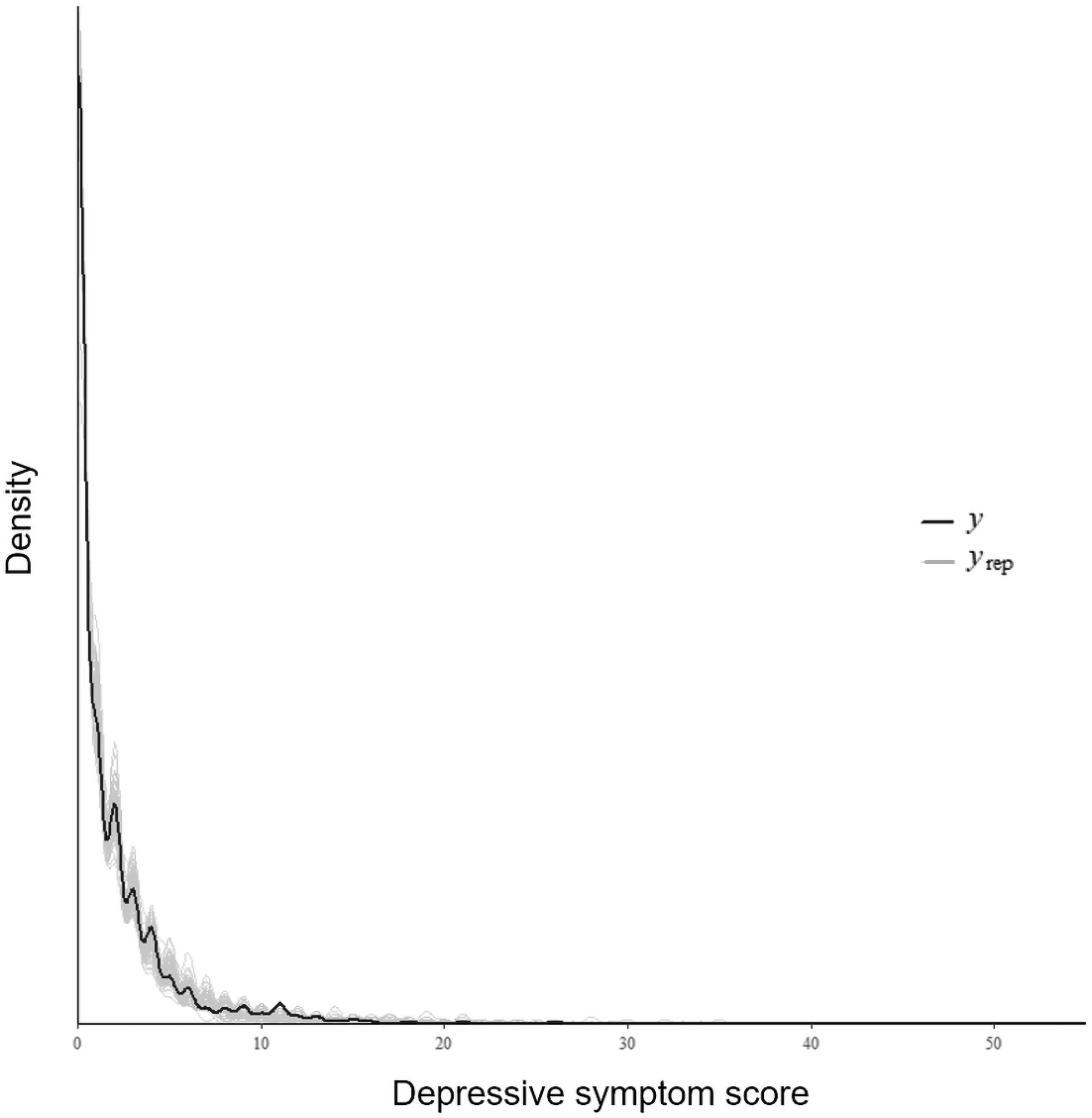
Posterior predictive checks of the Bayesian multilevel hurdle model. This plot indicates how well the fitted model can reproduce the observed data. A black, thick, solid line indicates the outcome distribution from the observed data. Solid grey lines indicate simulated outcome distributions generated from replicated data based on the fitted model. Y: outcomes from the observed data; Yrep: outcomes from the replicated data.

**Figure 4.**
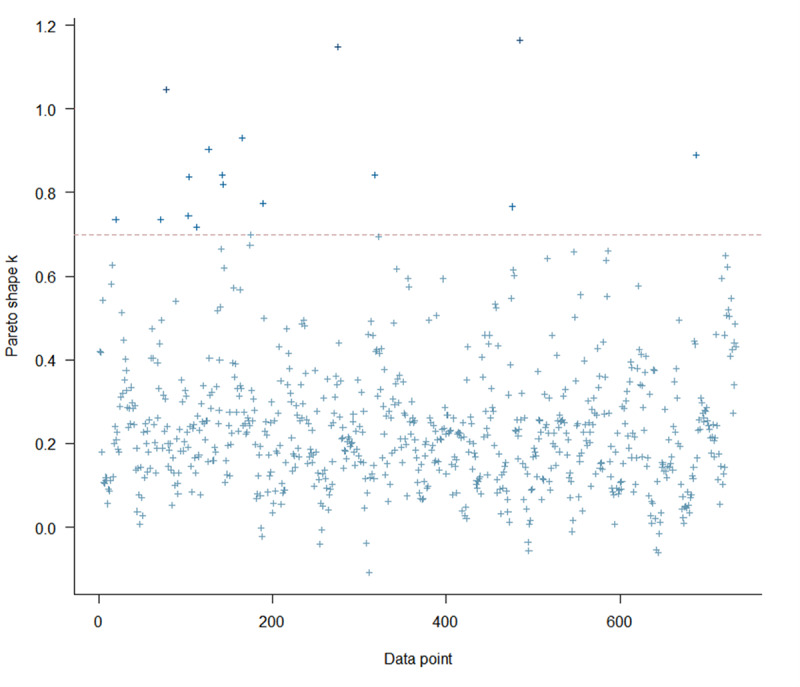
Pareto smoothed importance sampling (PSIS) diagnostic plot. This plot shows the reliability of evaluating a Bayesian model using leave-one-out cross-validation. Light blue cross marks indicate Pareto k values for each observation. Pareto k values under 0.5 are interpreted as very reliable importance sampling, and Pareto k values under 0.7 are regarded as acceptable.

As shown in Figure 3, the posterior predictive checks indicated that the Bayesian multilevel hurdle model adequately reproduced the observed distribution, including the mass at zero and the variability among nonzero values. Figure 4 demonstrates the Pareto smoothed importance sampling diagnostic test results of the model. When we examined the Pareto smoothed importance sampling diagnostic test results, most observations (97.8%) showed Pareto k values below 0.7, indicating a reliable leave-one-out cross-validation estimate (expected log predictive density calculated via leave-one-out cross-validation=−946.0; SE 35.5).

**Table 4.** Performance metrics of the Bayesian multilevel hurdle model.

Performance metrics	Training set	Test set
Continuous part		
RMSE^a^	1.60	2.25
MAE^b^	0.95	1.22
*R*^2c^	0.65	0.53
Calibration slope (95% CrI^d^)	—^e^	1.35 (1.06 to 1.64)
Calibration intercept (95% CrI)	—	0.32 (–0.13 to 0.77)
Binary part		
AUC-ROC^f^	0.95	0.88
Accuracy	0.87	0.79
Sensitivity (recall)	0.88	0.75
Specificity	0.87	0.82
Precision (PPV^g^)	0.86	0.76
*F*_1_-score	0.87	0.75
Brier score	—	0.14

a RMSE: root-mean-square error.

b MAE: mean absolute error.

c *R*2: coefficient of determination.

d CrI: credible interval.

e Not applicable.

f AUC-ROC: area under the receiver operating characteristic curve.

g PPV: positive predictive value.

## Discussion

### Principal Findings

This study has investigated how digital phenotyping data collected from wearable devices, including smartphones and smartwatches, can help monitor depressive symptoms in older adults. The major findings of this study provide empirical evidence about the use of digital phenotyping data for older adults’ mental health, as previous conceptual framework studies have suggested [26,27]. This study supports promising clinical implications for reducing the proportion of undiagnosed and untreated depressive symptoms.

Specifically, our results may expand the age range for which digital phenotyping can be used to monitor depressive symptoms. Our results demonstrate that including active and passive sensing digital phenotyping data alongside traditional in-person screening tools can enhance model performance, particularly in screening for total depressive symptom scores in older adults. In this study, the Bayesian multilevel hurdle model explains approximately 53% of the variance in depressive symptom severity (*R*^2^=0.53) and performs well (AUC-ROC=0.88; *F*_1_-score=0.75) in screening for the probability of experiencing any depressive symptoms among community-dwelling older adults. Machine learning is expected to be a valuable tool to screen for depression using a smartphone [28]. As the young generation rapidly acquires cutting-edge technology, previous research on digital phenotyping has primarily focused on college students [29]. However, given that help-seeking is unlikely to happen when taboos exist related to mental health problems [30] and smart devices are designed to be used intuitively, it can be helpful for older adults. Older adults are less likely to approach professional mental health services since they have spent most of their lifetime with strong taboos and stigmas about mental health than younger generations. Our findings from the Bayesian multilevel machine learning approach support the applicability of active and passive sensing digital phenotyping data to make professional mental health surveillance efforts more accessible to older adults.

This study also suggests considering the multilevel structure of the data. Recently, there has been an increase in academic attention to developing machine learning algorithms; many attempts treat multimodal data as a single dimension, focusing on the roles of passive sensing data [31,32]. Also, previous research reporting empirical evidence has tended to collect data over a short period [29]. However, when applying digital phenotyping to monitor depressive symptoms, it would be more beneficial to use longitudinal monitoring within the target population [33]. From a statistical perspective, data collected through longitudinal monitoring are nested data, with repeatedly collected data from the same individuals sharing characteristics at the person level. Thus, it violates the assumption of independence in simple linear or logistic regression. In this case, using a multilevel approach is necessary. So far, there has been little effort to apply a multilevel approach to machine learning models for continuous screening for depressive symptoms. The findings of this study show the potential applicability of multilevel modeling in developing machine learning models to screen for depressive symptoms. Interestingly, when we tested and compared the simple regression model vs the multilevel regression model results by using the same 6 PCs to explain the total score of depressive symptoms to handle our nested data, we saw that the Bayesian multilevel regression model results have a significantly higher *R*^2^ than the Bayesian regression model (*R*^2^ for test data: 0.32 in Bayesian regression → 0.52 in Bayesian multilevel regression). It implies that thoughtful consideration of multilevel data structures would enhance model performance.

When screening for depressive symptoms in the general population, there is heterogeneity between those who have a consistently low susceptibility to depression and those who are vulnerable to depression [12-14]. In this study, we also observed excessive zeros in our data. We used a Bayesian multilevel hurdle model to handle this. The results of this study demonstrate that using a Bayesian multilevel hurdle model can achieve good performance. As shown in Figure 2, posterior predictive checks indicated good agreement between the observed and simulated data generated from the posterior predictive distribution. This shows that the model adequately captured the key distributional features of the outcomes. Also, as shown in Figure 3, PSIS-LOO diagnostics demonstrated that most Pareto shape parameters (k) were below 0.7 (97.8%), with only a few values exceeding 0.7. This suggests stable importance weights and reliable estimation of out-of-sample predictive accuracy. These results support the adequacy and robustness of our proposed model.

When applying digital phenotyping to real-time monitoring to screen for depressive symptoms, a large number of features will be collected via multimodal approaches. To reduce dimensionality, researchers may use feature extraction or feature selection to handle the large number of features, unless they obtain funding and resources to collect large-scale data from a huge number of people. To screen for depressive symptoms, we used PCA-based feature extraction over feature selection. Given that the primary goal of screening for depressive symptoms using digital phenotyping is to promptly identify individuals who may be experiencing depressive symptoms and to approach them for early intervention, we think PCA-based feature extraction has the advantage of flexibly identifying specific profiles within those experiencing depressive symptoms residing in that community at that moment of monitoring. The mechanisms underlying depressive symptoms are not a single one [34], and thus using feature selection can introduce additional bias in explaining the targeted outcome, especially when the sample is modest in size or its representativeness is uncertain. Thus, we preferred feature extraction over feature selection, assuming it would lose less information. This study demonstrates interesting results from PCA and Bayesian modeling. For example, in the PCA results, the top 5 features for PC2 and those for PC4 appeared similar, but the rank of features and the directions of their factor loadings were shown differently in each latent PC. In other words, either in PC2 or in PC4, male and smoking had positive factor loadings. However, sleep-related features (light sleep and deep sleep) had positive factor loadings in PC2, whereas sleep-related features (light sleep, REM sleep, and deep sleep) had all negative factor loadings in PC4. In the Bayesian modeling, PC4 was significantly associated with the severity of depressive symptoms, while PC2 was not. We do not insist that the same PCA results would appear in other data. Rather, this result suggests that feature extraction using PCA may enhance more flexible and scalable community-based screening for depressive symptoms in older adults by uncovering high-risk profiles.

When extracting 6 PCs, we included all the features rather than separating time-varying features and time-invariant features. This is mainly because latent profiles for those experiencing depressive symptoms may emerge from combinations of personal characteristics and time-varying features, as we discussed in the Results section. If we separate time-varying features from time-invariant features at the PCA stage, PCs extracted from fragmented data will have less power to explain the target outcomes. In the PCA results of this study (Table 2), each PC shows a mixture of traditional survey tools and at least one active or passive digital phenotyping feature among the top 5 features for that PC. When extracting the PCs from the large number of parameters, our results showed that not only well-known in-person survey items such as social support (MSPSS), loneliness (UCLA Loneliness), and anxiety (GAD-7), but also active digital phenotyping parameters such as weekly stress, and passive sensing digital phenotyping parameters such as sleep-related features mainly contributed. By pooling time-varying states and time-invariant personal characteristics, we hope the PCA captures the overall covariance structure of features that co-occur in real-world digital phenotyping contexts. Consequently, the extracted components should be interpreted as composite trait-state dimensions reflecting joint patterns, rather than as pure latent traits or pure dynamic factors.

In this study, the Bayesian multilevel hurdle model results align with previous research findings. In either the continuous or the binary part, PC1 was significantly associated with the outcome. In extracting PC1, anxiety, loneliness, daily negative mood, and weekly stress had positive factor loadings, while social support had a negative factor loading. In the Bayesian modeling, PC1 was positively associated with the severity of depressive symptoms in the continuous part. This PC1 was negatively associated with structural zeros in the binary part. These findings align with previous research demonstrating that older adults with low psychosocial well-being are more likely to experience the occurrence of depressive symptoms and greater severity [35-37]. In addition, PC4 was not statistically significant for structural zeros, but it was positively associated with the severity of depressive symptoms. This means that male older adults who smoke heavily and have shorter REM, light, and deep sleep are likely to experience higher levels of depressive symptoms. It reveals a latent profile with the risk of greater depression severity in this community. This finding aligns with previous research addressing a positive association between smoking and depression, especially in older adults [38]. This finding is also consistent with other research showing that greater wakefulness and reduced sleep efficiency (total time in bed minus awakened time) are associated with an increased risk of depression in male older adults [39].

In the binary part, the hurdle model results show a trend of increasing zeros over time. We were concerned that participants might experience fatigue when responding to the monthly PHQ-9 survey. When we tested whether the variance of responses to each PHQ-9 item decreased, no such trend was observed. When we examined average step counts and sleep efficiency between cases in which participants reported zero and those in which they reported nonzero, the average step counts (8512 in nonzeros and 9724 in zeros) and sleep efficiency (85.9% in nonzeros and 86.1% in zeros) were higher in the zero cases. These results suggest that the increase in zeros over the month in the binary part can be interpreted as a signal of symptom improvement. This aligns with previous research on the subjective experience of long-term remote monitoring of depressive symptoms using technology [40]. Through multisite, longitudinal qualitative interviews, participants reported participating in the study with an altruistic intention to help others by sharing their experiences, but they also experienced benefits, such as increased self-awareness. In our research, we also heard reports from some participants that their study engagement made them aware of their mood and more attentive to their health conditions. This implies that longitudinal monitoring using digital phenotyping itself can have clinical implications for enhancing older adults’ self-awareness and self-care in the community.

One thing to consider is that our results showed a random-intercept SD of 3.07 in the binary part, suggesting that baseline symptom propensity can inform a strong personal trait. By splitting the chronological data into 8:2 at the observational level for each participant, we allow our model to use known random intercepts for participants seen in the training data. As formalized in the multilevel logistic regression model equation [41], our result indicates that approximately 74% of the variance is found at the between-person level, while there remains substantial within-person variance (approximately 26%) available for modeling. In fact, this degree of heterogeneity is common in longitudinal psychiatric research, where individuals with few symptoms contribute to wide separation in intercepts [12-16]. On the logit scale, this variance reflects the high degree of person-level stability in behavioral health, yet it leaves sufficient within-person variance to be modeled by using digital phenotyping features. In a recent study [42], reanalyzing intraindividual consistency in secondary data by profiles of daily smartphone usage showed that within-person variance is better explained in personality research when accounting for between-person variance with longitudinal data collected from the same participants. Another previous research [43] underscores the benefits of digital phenotyping for capturing unique data streams for each individual. Although it is challenging to establish population-level patterns given the heterogeneity of symptoms across the total population, digital phenotyping is a valuable tool for within-person health tracking, helping capture individual malfunctions effectively and develop tailored intervention plans [43]. Taken together, digital phenotyping can be a useful tool for personalized, within-person health tracking, even after accounting for substantial between-person variance.

Additionally, this study’s findings suggest that handling data imbalance will be a critical challenge when screening for depressive symptoms in the general population, even after accounting for active and passive digital phenotyping and a multilevel data structure. Although major depressive disorder is prevalent, the absolute number of individuals experiencing depressive symptoms would be much smaller than that of nonsymptom individuals. According to the National Center for Mental Health in South Korea [44], for example, the annual prevalence of depressive disorder is 1.7%. In fact, regardless of the disease type, data imbalance has been observed and discussed in other medical studies. For example, a recent study [45] developing a predictive model for a physical disease found that a massive dataset helps address data scarcity, but it still faces the challenge of data imbalance. Despite the enormous volume of data, a severely skewed distribution persists, and the minority class remains underrepresented. As recent studies have been exploring [46-48], future research needs to consider not only the increase in data size but also resampling and synthetic data generation.

### Limitations

This study has several limitations, which require readers to exercise caution. First, due to the high cost of smartwatches, this study targeting older Korean adults analyzed data from a modest sample. Also, we were unable to install our smartphone app on an iOS device (Apple Inc) because of technical issues. Thankfully, more than 85% of older Korean adults aged 50 to 80 use Android (Google LLC) smartphones [49], so we proceeded with our research. However, future research would be better served by collecting data from mobile devices and smartwatches running both Android and iOS. In addition, given that the primary purpose of this research project was to reduce the risk of unrecognized depressive symptoms among older Korean adults, we targeted the general population. However, this led to a small proportion of clinical depression in our data. Although more research is needed to delve into interpersonal and intrapersonal differences in longitudinal patterns of depressive symptoms when applying digital phenotyping in practical settings [50], this limitation hinders testing more complex dynamics between digital phenotyping data and different types of depressive disorders. Collecting more clinical cases would enable comparison of the relationship with digital phenotyping across mood disorder types. This approach could help develop more personalized mental health surveillance in digital phenotyping research. Finally, in this study, the calibration slope for the continuous part in the Bayesian multilevel hurdle model was 1.35, suggesting that it tends to underestimate severity at lower predicted values, although the model effectively captures relative differences in symptom severity. In the context of digital phenotyping applications, such miscalibration may lead to inflated risk stratification and premature escalation of clinical interventions if raw predicted scores are used directly. While the model appears suitable for ranking individuals by relative symptom burden and supporting population-level screening, additional screening is necessary before applying predicted PHQ-9 scores to guide individualized clinical decisions. These results underscore the importance of explicitly evaluating and correcting calibration when deploying machine learning–based severity prediction models in real-world monitoring settings.

### Recommendations

Despite the limitations of this study, these findings can contribute to future research on mental health and digital phenotyping in related academic fields by providing empirical evidence from Bayesian multilevel machine learning models applied to older adults. Recently, there has been a rapid increase in protocol studies and review studies in digital phenotyping. It is recommended to gather additional empirical evidence to address challenges, replicate the findings, and explore more effective strategies to enhance the implementation of digital phenotyping for better screening of geriatric depression. Digital phenotyping, including active and passive data alongside traditional in-person screening tools, can help monitor depressive symptoms in older adults. Further investigation into the development and validation of effective digital biomarkers is also recommended to detect depression signals early in the general population.

### Conclusion

In this study, we investigated how digital phenotyping, including active and passive sensing data in addition to traditional in-person surveys, can help monitor depressive symptoms among older adults. To account for time-varying and time-invariant features, we analyzed the data using Bayesian multilevel modeling. Specifically, we used a Bayesian multilevel hurdle model to account for the data distribution reflecting heterogeneity in older adults’ experiences of depressive symptoms. Dimensional reduction using parallel analysis and PCA, covering all parameters collected from in-person, active, and passive data, can be helpful to reveal latent profiles with a high risk of depressive symptoms. A Bayesian multilevel hurdle model yields acceptable performance for depressive symptom severity and depressive symptom susceptibility for new data from participants. At least one active and one passive digital phenotyping feature were among the top 5 features for the major PCs. These results support the idea that digital phenotyping data help enable real-time monitoring to screen for depressive symptoms in the general population. Future research on effectively handling data imbalance would be beneficial for advancing academic research on digital phenotyping, particularly for monitoring depressive symptoms in community-dwelling older adults.

## Supplementary material

10.2196/69494 Multimedia Appendix 1 Descriptive statistics of the participants.

10.2196/69494 Multimedia Appendix 2 Logistic regression results for attrition bias.

## Abbreviations

AUC-ROC: area under the receiver operating characteristic curve
CrI: credible interval
ESS: effective sample size
ETI-SF: 27-item Early Trauma Inventory–Short Form
GAD-7: 7-item Generalized Anxiety Disorder
MAE: mean absolute error
MICE: Multiple Imputation by Chained Equations
MINI: Mini-International Neuropsychiatric Interview
MSPSS: Multidimensional Scale of Perceived Social Support
PC: principal component
PCA: principal component analysis
PHQ-9: 9-item Patient Health Questionnaire
PSIS-LOO: Pareto smoothed importance sampling leave-one-out
REM: rapid eye movement
RMSE: root-mean-square error
UCLA Loneliness: University of California, Los Angeles Loneliness Scale
