# Supplementary material for: Using Digital Phenotyping for Depression Screening in Community-Dwelling Older Adults: Bayesian Multilevel Hurdle Model Machine Learning Approach
Source: JMIR AI. 2026 May 15;5:e69494. doi: 10.2196/69494 (PMC13179049; doi:10.2196/69494)
Supplement: Multimedia Appendix 1 [file ai-v5-e69494-s001.docx]

Appendix 1. Descriptive statistics of the participants

|  | Total participants who completed PHQ-9 and traditional in-person surveys  (N = 352) | Participants who had active and passive digital phenotyping data  (N = 147) |
| --- | --- | --- |
|  | N (%) or M (SD) | N (%) or M (SD) |
| Sex |  |  |
| -Female | 203 (57.67%) | 85 (57.82%) |
| -Male | 149 (42.33%) | 62 (42.18%) |
| Age | 67.23 (6.53) | 67.08 (6.12) |
| Education |  |  |
| -Less than a high school degree | 131 (37.22%) | 52 (35.37%) |
| -High school degree | 126 (35.80%) | 60 (40.82%) |
| -Bachelor’s degree | 76 (21.59%) | 28 (19.05%) |
| -Graduate school degree | 19 (05.40%) | 7 (04.76%) |
| Major depressive history |  |  |
| -No | 317 (90.06%) | 129 (87.76%) |
| -Yes | 35 (09.94%) | 18 (12.24%) |
| Freq. of depressive episodes | 0.69 (1.66) | 0.67 (1.60) |
| Household income (log) | 14.78 (0.67) | 14.82 (0.65) |
| Agricultural job |  |  |
| -No | 312 (88.64%) | 127 (86.39%) |
| -Yes | 40 (11.36%) | 20 (13.61%) |
| Married |  |  |
| -No | 29 (08.24%) | 14 (09.52%) |
| -Yes | 323 (91.76%) | 133 (90.48%) |
| Number of family members | 2.36 (0.88) | 2.31 (0.81) |
| Number of chronic diseases | 1.16 (1.14) | 1.21 (1.19) |
| Regular exercise |  |  |
| -No | 144 (40.91%) | 58 (39.46%) |
| -Yes | 208 (59.09%) | 89 (60.54%) |
| Daily sleep hours in total | 6.46 (1.31) | 6.54 (1.30) |
| Perceived social support | 3.98 (0.69) | 3.97 (0.74) |
| Loneliness | 1.75 (0.45) | 1.71 (0.48) |
| Generalized anxiety disorder | 1.38 (2.74) | 1.48 (2.73) |
| Early childhood experiences | 2.95 (2.96) | 2.90 (2.70) |
| Drinking (cups per month) | 14.53 (33.41) | 14.35 (34.82) |
| Smoking history |  |  |
| -No | 239 (67.90%) | 104 (70.75%) |
| -Yes | 113 (32.10%) | 43 (29.25%) |
