# Supplementary material for: Using Digital Phenotyping for Depression Screening in Community-Dwelling Older Adults: Bayesian Multilevel Hurdle Model Machine Learning Approach
Source: JMIR AI. 2026 May 15;5:e69494. doi: 10.2196/69494 (PMC13179049; doi:10.2196/69494)
Supplement: Multimedia Appendix 2 [file ai-v5-e69494-s002.docx]

Appendix 2. Logistic regression results for attrition bias

|  | Attrition for passive  digital phenotyping data  Dropped (1) vs. Included (0) | |
| --- | --- | --- |
|  | OR (S.E.) | 95% CIs |
| Male | 0.68 (0.25) | 0.33, 1.40 |
| Age | 1.01 (0.02) | 0.97, 1.05 |
| Education | 1.08 (0.16) | 0.81, 1.45 |
| Major depressive disorder history | 0.48 (0.20) | 0.21, 1.09 |
| Number of depressive episodes | 1.08 (0.09) | 0.91, 1.29 |
| Monthly income | 0.76 (0.16) | 0.51, 1.14 |
| Agricultural job | 0.63 (0.23) | 0.30, 1.29 |
| Married | 1.55 (0.68) | 0.66, 3.65 |
| Number of family members | 1.10 (0.15) | 0.84, 1.44 |
| Number of physical chronic diseases | 0.92 (0.10) | 0.75, 1.13 |
| Regular exercise | 0.86 (0.20) | 0.54, 1.37 |
| Total sleep hours | 0.95 (0.09) | 0.80, 1.13 |
| Perceived social support | 1.49 (0.35) | 0.95, 2.35 |
| Loneliness | 1.91 (0.70) | 0.93, 3.94 |
| Generalized anxiety disorder | 0.95 (0.05) | 0.86, 1.04 |
| Adverse childhood experiences | 1.01 (0.04) | 0.93, 1.10 |
| Drinking | 1.00 (0.00) | 0.99, 1.01 |
| Smoking history | 1.63 (0.59) | 0.80, 3.31 |
